# The role of cognitive and non-cognitive factors in mathematics achievement: The importance of the quality of the student-teacher relationship in middle school

**DOI:** 10.1371/journal.pone.0231381

**Published:** 2020-04-20

**Authors:** Cristina Semeraro, David Giofrè, Gabrielle Coppola, Daniela Lucangeli, Rosalinda Cassibba

**Affiliations:** 1 Department of Education, Psychology, Communication, University of Bari Aldo Moro, Bari, Italy; 2 Department of Education, DISFOR University of Genoa, Genoa, Italy; 3 Department of Developmental Psychology and Socialization, University of Padova, Padova, Italy; University of Westminster, UNITED KINGDOM

## Abstract

There is increasing evidence that several factors, including both cognitive and non-cognitive ones, play an important role in mathematics achievement. Relatively little is known about how socio-emotional features and the quality of the student-teacher relationship correlate with mathematics achievement among adolescents in transition to middle school. The aim of the present study is to examine the role of cognitive factors (general cognitive abilities), non-cognitive factors (math anxiety and self-esteem), and the quality of the student-teacher relationship on mathematics achievement. A large sample of Italian sixth graders was evaluated upon entering middle school. The results showed that general cognitive ability was the best predictor of mathematics achievement. As regards non-cognitive factors, the level of math anxiety was effective in predicting mathematics achievement, after controlling for other measures including self-esteem and the quality of the student-teacher relationship. In particular, we found that the quality of the student-teacher relationship had an indirect influence on mathematics achievement through the mediation of math anxiety. Our findings seem to indicate that the quality of the student-teacher relationship may be related to mathematics achievement, through its effects on math anxiety. This may have important implications for practitioners and educators, as we can suggest that interventions devoted to improving the quality of the student-teacher relationship may play a positive role in both preventing math anxiety and promoting mathematics learning.

Solving mathematical tasks is a complex task that involves several distinct abilities that are essential in everyday life situations. Therefore, understanding the factors related to strong mathematical abilities is extremely important. Research has primarily focused on the influences of many individual factors, both cognitive and non-cognitive ones [1–3], and recent studies highlight the complexity of the interaction between them [4–6]. More recent contributions have expanded the focus beyond the individual level, and have targeted factors allocated on the broader environmental level, such as the classroom, the relationship with the teachers and the family context [7–11]. There is now a general consensus that a deep understanding of mathematics achievement requires research attention of both individual and environmental factors and how these factors might interact [7–9]. With this respect, Chang and Beilock (2016) propose a conceptual framework which attempts to integrate these influences in order to explain the mathematics achievement gap existing among student. Although this theoretical model is extremely interesting, as it provides a framework from which to consider the multiple factors implicated in math performance, empirical evidence supporting the model is still scant.

As to the individual level, among the cognitive factors, general cognitive ability involves the ability to reason, plan, solve abstract problems, think abstractly, comprehend complex ideas, and maintain and manipulate complex materials [12]. The construct of general intelligence or general cognitive abilities (usually denoted as g) is a quantitative trait, and various theories of intelligence have attempted to measure and quantify human intellectual abilities [13]. Among the many contemporary intelligence theories, the Cattell–Horn–Carroll (CHC) theory is a most influential one that offers an interpretation of the relationship between intelligence and academic achievement [14, 15]. Specifically, the CHC theory of intelligence is a synthesis of Cattell and Horn’s theory of fluid and crystallized intelligence [16–18] and Carroll’s Three-Stratum model [15, 19]. There is extensive literature demonstrating that general cognitive abilities and mathematics achievement are strongly associated [20, 21]. The fact that general cognitive abilities are strongly related to mathematical outcomes is not surprising. The understanding of mathematical task esteems requires the formation of abstract representations of quantitative and qualitative relationships between variables [22]. Further, solving mathematical tasks requires the ability to link second-order relationships in a logical and ordered manner and the ability to manipulate visual representations [23]. In particular, general cognitive abilities seem to be involved in complex mathematical skills required in middle school age. For example, solving mathematics problems involves reasoning skills, as they are needed to: (a) construct a structure coherent with the salient information of the text of the problem; (b) integrate information from the text with inferences based on the child’s knowledge (fluid and crystallized intelligence); and, (c) create schema to formalize the relationships among quantities and guide the application of solution strategies [24]. Moreover, other complex mathematics tasks in middle school mathematics curricula, such as fractions and algebra, are built on foundational numerical and calculation skills, which are thought to require general cognitive abilities to a large degree [25–28].

Besides general cognitive ability, which has been consistently related to mathematics achievement [20], also non-cognitive factors seem to play an additional role and, among these, math anxiety [29–31]: this construct can be broadly defined as a state of discomfort caused by performing mathematical tasks and expresses itself as a feeling of fear that many people experience when engaging in mathematical tasks [32–34]. In fact, math anxiety is a multivariate construct that includes: math learning anxiety, which refers to feelings of tension when content has to be learned; math evaluation anxiety, which refers to situations in which performance in mathematics is being evaluated; and, finally, generalized school anxiety, as to an extension of anxiety to other school subject [35]. The relationship between math anxiety and mathematics could be interpreted within the theoretical framework of reduced competency account, which argues that math anxiety is actually the result of poor math ability [35]. In this theory, a student’s poor skill leads to low competence in mathematics achievement, which then contribute to raise levels math anxiety. This theoretical approach is supported by the studies of Maloney and colleagues [35]. These Authors showed that individuals starting with lower numerical skills tend to underperform in math. As an effect of this underperformance, they become anxious. The consequence of this state of anxiety towards mathematics tasks is that students already with lower math skills, also tend to avoid studying this discipline. In fact, the typical behaviors of these students are avoidance of math homework or low participation in classroom activities [36]. These avoidant behaviours lead to further delays in learning and consequently higher levels of anxiety. Other studies have in fact showed that math anxiety, in particular among adolescent students, was connected to a scarce attendance of mathematics courses [35–36]. Math anxiety might be particularly interesting to investigate in middle school age, as the evidence shows that it might increase as student move to middle school [37].

Besides math anxiety, another non-cognitive factor, self-esteem, appears to be related to mathematics achievement [38–42]). Self-esteem is definable as “an individual’s subjective evaluation of his or her worth as a person” (Orth & Robins, 2014, p. 381), and includes evaluations of the self along a “good–bad” dimension [43, 44]. Self-esteem requires a higher-order integration of domain-specific attributes which occurs only in early adolescence; young children do not begin to describe themselves in terms of concrete cognitive abilities, physical abilities, how they behave, how they look, and friendships they have formed. Indeed, in young children these domains are not clearly differentiated from one another, as revealed through factor-analytic procedures, nor are they integrated into a higher-order concept of self-esteem [45]. For these reasons, adolescence seems to be the appropriate age to explore this construct and its impact on academic achievement. The concept of self-esteem and its relation to academic achievement is controversial in the literature, especially with respect to the age group we are interested in [43–46]. In their review, Baumeister et al. (2003, pp. 13–14) concluded that, “*the few studies suggesting any positive causal impact of self-esteem [on academic achievement] generally found only tiny effects*. *Some findings even point (again weakly) in the opposite direction*, *suggesting that high self-esteem may detract from subsequent performance*”. Conversely, other studies suggest that high self-esteem is an important factor to consider in the prediction of academic achievement in students [46–51]. In their work, Alves-Martins and colleagues (2010) showed that students with lower levels of school success attach less importance to school subjects and show poorly favorable attitudes towards school [52]. More clarifications come from a longitudinal study conducted by Trautwein et al. (2006), who examined the dynamics between mathematics achievement, self-concept, and self-esteem [53]. These scholars showed that high self-esteem is not a strong predictor of later achievement but, when they restricted their analyses to the reciprocal relationship between self-esteem and mathematics achievement, they found some small but significant predictive effect on mathematics achievement. In particular, the effects of self-esteem on mathematics achievement seem to be mediated by self-concepts. In sum, it is difficult to draw firm causal conclusions about the effects of self-esteem on mathematics achievement. There is no strong evidence specifically indicating that high self-esteem leads to improved mathematics achievement, or that better mathematics achievement leads to better self-esteem. It is precisely this controversial nature of the relationship between self-esteem and mathematics that led us to investigate the effects of this construct on mathematics achievement.

Other studies additionally explored the relation between self-esteem and math anxiety [54–57]. Existing findings support reciprocal influences between the two domains in middle school age. In particular, the study by Akin and Kurbanoglu (2011) showed that self-esteem predicted math anxiety directly as well as through a pathway mediated by the positive or negative attitudes to mathematics. Conversely, through a longitudinal design, Ahmed and colleagues (2012) showed that lower self-esteem levels predicted higher levels of math anxiety. At the same time, higher levels of math anxiety predicted lower levels of self-esteem, by controlling for previous levels of self-concept, supporting reciprocal influences between the two domains over time [58]. Finally, a recent study by Xie and colleagues (2019) explored the relationship between self-esteem, math anxiety and mathematics achievement in a sample of students between 12 and 18 years old. The researchers showed, through structural equation models, how self-esteem was able to directly predict math anxiety but not mathematics achievement in male students, while in female students, self-esteem predicted neither math anxiety nor much less mathematics achievement [59]. In concise, given the complex nature of the construct and the controversial relation between self-esteem, math anxiety and mathematics achievement, we found it useful to bring new knowledge to literature.

Whether some consensus exists on the impact of general cognitive abilities, math anxiety, and self-esteem on mathematics achievement, the relations between these different constructs has not been addressed so far. To the best of our knowledge, only one study on sixth graders has explored jointly two of the three suggested predictors (intelligence and self-esteem) and their impact on mathematics performance [20]. Giofrè, Borella, and Mammarella (2017) showed that intelligence was the best predictor of mathematics achievement, but also self-esteem was able to predict mathematics achievement. The Authors showed that their data fitted best a model including a direct link from intelligence to the mathematics achievement and direct and indirect effects of self-esteem on mathematics achievement through intelligence. The researchers speculated that students with higher cognitive abilities also tended to have higher levels of self-esteem, which in turn led to higher mathematics performance.

As to the contextual factors which might impact mathematics achievement, recent research has stressed the importance of the quality of the student-teacher relationship (e.g., [60, 61]). For the last two decades, the quality of the student–teacher relationship has been a focus of educational research [62]. This line of study is rooted in attachment theory [63], which provides a theoretical framework for understanding the relevance of a caregiver’s sensitivity to children’s cues as a prerequisite for secure relationships. In applying this concept to relationships in the classroom, thanks to Robert Pianta’s work [64], empirical attention to the relationships with teachers has been shifted from the educational dimension to the affective one. Specifically, Pianta (1999) proposed the conceptualization of the relationship between teachers and students as an attachment relationship: as such, it will function as a secure base for the student to explore new learning opportunities, as well as a safe haven in which to regulate negative emotions within the school context. Since this conceptualization was shared in the academic community, a great amount of research has been conducted in order to provide empirical support to the theoretical idea according to which a positive affective relationship with a teacher might promote learning and positive adaptation within the school context [62] and the affective quality of the student–teacher relationship has been shown to be an important predictor of children’s development and wellbeing [65]. Many studies have found that students with close relationships with their teachers are more likely to experience at school academic interest, engagement, achievement, self-efficacy, and motivation as compared to students with more distant relationships [66–78]. Particularly for children who are at risk of failure in school, an emotionally supportive relationship with a teacher can act as a protective factor and have positive effects on developmental outcomes [66]. Furthermore, a positive teacher-student relationship would allow students with difficulties in learning new or complex content to approach the teacher easily, which increases the possibility of the teacher adapting instruction to favour the consolidation of academic performances [79]. One gap exists in this research are, as most studies have focused on the pre-school age group [80–83] and on the primary school age group [84–86], while early adolescence and the transition to middle school still seems scarcely explored. Considering this state of the art, we suggest that this age deserves more research attention.

Notwithstanding the relevance of cognitive (i.e. general cognitive abilities) and non-cognitive factors (i.e. math anxiety and self-esteem), at the individual level, as well as of the quality of student-teacher relationship, at the contextual one, on mathematics achievement, this far there is no empirical test of their joint influence in the transition to middle school. We selected this age group because general cognitive ability seems to be relatively stable from this age on [20, 87]. In addition, at these ages the complex interactions between cognitive and non-cognitive factors is influenced by the complexity of the mathematical task and by the increase in the required cognitive abilities [87]. In fact, middle school seems to be a critical moment in which to evaluate this interaction [87–90]. We decided to use a latent modelling approach because it presents several advantages (e.g., being more precise and reliable) as compared to traditional analyses, and it has been successfully used by researchers in this area (e.g. [20, 91, 92]).

## Method

### Participants

The initial sample consisted of 219 children attending the sixth grade. Children with clinical diagnoses and those belonging to disadvantaged socio-cultural groups were not included in the study (4 children). Children who were absent for at least one session were excluded from the analysis (30 children). Using casewise deletion with Mehalanobis distance, some participants (n = 4) were found to be multivariate outliers (D > 40) and were excluded from the analyses. Therefore, the final sample included 181 children (females = 49%, average age = 10.65, SD = 0.49). The children were attending school in an urban area of Puglia, in Southern Italy. All information at the item level was available for these children.

### Ethics statement

The study had the prior approval of the Local Ethics Committee of the Department of Education, Psychology, Communication at the University of Bari. All the parents provided written informed consent on behalf of their children prior to their participation in the study.

### Instruments

#### General cognitive abilities (g)

*Cattell culture fair intelligence test* [93]. The task consisted of four timed subtests (series completion, odd-one-out, matrices, and topology) with items of increasing difficulty within each subtest. The test contained 46 multiple choice items (KR-20 = .66). The score was the sum of the correct answers. The CFIT is a well-known matrix reasoning instrument assumed to be independent from cultural experiences. It includes two equivalent forms (A and B) and four subtests involving multiple-choice problems progressing in difficulty: series (8 items), classification (14 items), matrices (12 items), and conditions (8 items). The combined form A + B is mainly used in a clinical setting, while in the school setting, form A is preferred. Thus, for our purposes, we considered form A. The scores ranged from 0 to 46. The validity of the CFIT has long been established [94] and the subtest scores are usually taken to be strong indicators of one comprehensive latent construct of fluid intelligence.

*Primary Mental Abilities–Spatial* (PMA-S; [95]). In this task, the subject was required to identify, among six different rotated figures, the figures that were exactly alike: i.e. figures that were rotated but mirrored. The test consisted of 20 trials with two or more correct alternatives for each (KR-20 = .89). The score was the sum of the correct answers.

*Primary Mental Abilities–Reasoning* (PMA-R; [95]). In this paper-pencil test, the child had to complete a sequence of letters, choosing which letter from various alternatives logically completed the series (for example: in the series *abm*, *cdm*, *efm and ghm*, the answer is *i*). The test included 30 items and had to be completed within 5 minutes (KR-20 = .85). The score was the sum of the correct answers.

*Primary Mental Abilities–Verbal* (PMA-V; [95]). In this paper-pencil test, the children had to choose a synonym for a given word from among four options; for example, for the item, *small*: (a) *slow*, (b) *cold*, (c) *simple*, and (d) *tiny*, the answer is (d) *tiny*. There was only one correct answer for each question. The test included 50 items and had to be completed within 4 minutes. The score was the sum of the correct answers (KR-20 = .85).

The *visual pattern test–active task* (VPTA; [96–97]) tests the ability to maintain and process spatial information simultaneously presented on a digital blackboard in the classroom. A total of 18 matrices (adapted from the visual pattern test [98]) of increasing size (the smallest with 4 squares and 2 cells filled and the largest with 14 squares and 7 cells filled) contained a variable number of cells to remember (from 2 to 7). After each matrix had been shown for 3 seconds, the children were presented with a blank test matrix on which they were asked to reproduce the pattern of the previously seen cells by clicking in the cells corresponding to the same positions but one row lower (the bottom row in the presentation matrix was always empty). The score was the number of accurately placed cells.

#### Math anxiety

*Metacognition in Mathematics* (*MeMa*) [99] is composed of three different questionnaires that evaluate attitudes toward math. The first questionnaire is addressed to the children (from 8 to 14 years old), the second one is addressed to the primary and secondary school teachers, and the third one is for the parents. The questionnaire measures the level of math anxiety in students aged 8 to 14. For the purpose of this study, we only used the last questionnaire. It is composed of Likert Scale items and analyzes three different factors: *Mathematical Learning Anxiety* (16 items), *Mathematical Evaluation Anxiety* (8 items), and *School Generalized Anxiety* (6 items). Responses to these items are given on a 4-point scale (1 –low anxiety, to 4 –high anxiety; Cronbach’s *α* = .91).

#### Self-esteem

*Multidimensional Self-Concept scale* (MSC; [100]). This self-reported scale aimed to evaluate children’s feelings about themselves in six different domains of life, each assessed by a specific subscale which can be used separately. For the purposes of the study, three subscales were selected to assess the children’s feelings about themselves in terms of their interpersonal relationships, emotional competencies, and general life situations. Each subscale consisted of 25 positive and negative sentences for which respondents were asked to rate their agreement on a 4-point Likert Scale, from “absolutely true” to “absolutely false”. The test confirmed its good psychometric properties (quality of interpersonal relationships, Cronbach’s *α* = .87; emotional competencies, Cronbach’s *α* = .87; environment capability, Cronbach’s *α* = .79). The selection of the scales for assessing self-esteem was led by identifying the relevant measures by emotional and relational dimensions, which immediately guided our study. In this perspective, only three scales of the MSC Test tool were in line with our objectives: (a) the scale of interpersonal relationships (examples of items: “people like to be with me”; “others avoid me”; “I often feel left out”) allowed us to explore the dimension of relationships, a crucial aspect in our work; (b) the scale of control over the environment (examples of items: “I have confidence in myself”, “I am not very intelligent”, etc.) allowed us to measure the student's belief systems. In this perspective, some studies have highlighted the relevance of these aspects on the student’s self-esteem [101, 102]; finally, (c) the emotional scale represents a measure of the student's emotional life. This measure seemed extremely useful in terms of the ability to recognize and manage emotions and to control negative emotions. The other scales of the instrument allowed us to detect measurements such as: (d) family life and relationships in the family and the degree to which the student feels loved and valued; (e) body intended as a physical aspect, physical and sporting abilities. Finally, we consciously decided not to focus our attention on the dimension of (f) academic self-concept, since the effects of this construct on academic learning have been widely covered by the literature [103–110]. What instead seems to still be debated and is, consequently, more interesting is the effect of more general constructs such as self-esteem on academic learning [44, 45].

#### Student-teacher relationship quality

*Student-Teacher Relationship Questionnaire* (STRQ). An Italian adaptation [111] of the Student-Teacher Relationship Questionnaire [112] was used. The instrument examines children’s perceived relationships with teachers, as well as their generalized perceptions of the overall school environment. Based on attachment theory [63, 113], all the questions are designed to assess the positive and negative affective and cognitive experiences of warmth, trust, accessibility, and responsiveness within these relationships [114]. School bonding questions were developed based on prior theory and research in the area of social bonding [115] and were designed to measure positive bonding experiences. The final questionnaire was composed of 19 items: *Affiliation with Teacher* (Cronbach’s *α* = .88)– 8 items, *Dissatisfaction with Teacher* (Cronbach’s *α* = .66)– 3 items, and *Bonds with School* (Cronbach’s *α* = .80)– 8 items. Responses to this instrument were given on a 4-point scale (1—almost never or never true to 4—almost always or always true).

#### Mathematics achievement

*Achievement in Mathematics* (AC-MT 11–14) [116]. All students were administered the Italian AC-MT 11–14 Test by means of a set of paper-and-pencil tasks that can be grouped into three areas: written calculation, mathematical reasoning, and number knowledge. As described in the AC-MT Test manual, all subtests had standardized measures, showing a high level of reliability (Cronbach’s *α* = .80). *Written calculation* tasks assessed the use of arithmetic procedures, arising from knowledge of a body of rules (i.e., correct manipulation of numbers and operations, and rapid retrieval of results). *Mathematical reasoning* evaluates calculation speed and the student’s ability to manipulate large numbers (approximate calculation) and reasoning ability based on flexible use of basic calculation procedures and strategies (facts retrieval). *Number knowledge* tasks assess the components of calculation. Written calculation tasks assess the use of arithmetic procedures, arising from the knowledge of a body of rules (i.e., correct manipulation of numbers and operations, as well as rapid retrieval of results). Number knowledge tasks assess the components of calculation (e.g., number magnitude judgments). Mathematical reasoning tasks assess calculation speed, the student’s ability to manipulate large numbers (e.g., approximate calculation), and reasoning ability based on flexible use of basic calculation procedures and strategies (e.g., facts retrieval). The scores for the entire test ranged from 0 to 60. The Spearman-Brown split-half reliability coefficient in this study was .76 for the entirety of measurement.

### Procedure

Selection of the tasks was based on agreements with the schools participating in the study. In particular, the tasks were administered as part of a broad study promoted by the Department of Education, Psychology, Communication at the University of Bari Aldo Moro in Italy regarding the relation between academic achievement and teaching methods and strategies. The children were tested in October, one month after the start of school, in three collective sessions. Students were tested in different phases: (a) two group sessions in their classroom that lasted approximately one hour each: In the first group session, General Cognitive Abilities and Math Anxiety measures were assessed, while in the second group session Multidimensional Self-concept Scale and Student-Teacher Relationship Questionnaire measures were assessed; and (b) one group session in which we assessed mathematics achievement. During the first group session, the intelligence tests and the math anxiety questionnaire were administered in a fixed order (Cattell, PMA-V, PMA-S, PMA-R, VPTA and MeMa Test); in the second group session, the self-esteem subscales (quality of interpersonal relationships, emotional competencies, and environment capability) were also administered in a fixed order.

### Data analytic approach

The R program [117] with the “psych” [118] and “lavaan” [119] libraries were used to perform exploratory and confirmatory analyses. Model fit was assessed using various indexes according to the criteria suggested by Hu and Bentler (1999) [120]. We considered the chi-square (*χ*^*2*^), the comparative fit index (*CFI*), the non-normed fit index (*NNFI*), the standardized root mean square residual (*SRMR*), and the root mean square error of approximation (*RMSEA*); the chi-square difference (*Δχ*^*2*^), and the Akaike information criterion (*AIC*) were also used to compare the fit of alternative models [121].

## Results

The principal descriptive statistics and the correlation matrix are reported in Table 1.

**Table 1:** Correlations, means (M), and standard deviations (SD), for all measures.

	1	2	3	4	5	6	7	8	9	10	11	12	13	14	15	16	17
01. CAT	1																
02. PMA-S	.33	1															
03. PMA-R	.43	.29	1														
04. PMA-V	.36	.13	.33	1													
05. VPTA	.43	.37	.35	.18	1												
06. MA-MLA	-.15	.10	-.19	-.16	-.09	1											
07. MA-MEA	-.18	-.07	-.19	-.16	-.18	.69	1										
08. MA-SGA	-.03	.03	-.14	-.15	.09	.49	.46	1									
09. MCS-A	.01	-.06	.08	-.01	-.05	-.16	-.11	.00	1								
10. MCS -B	.06	-.02	.09	.11	-.08	-.22	-.19	-.18	.65	1							
11. MCS -C	.05	-.06	.09	.15	-.02	-.24	-.27	-.14	.63	.70	1						
12. QSTR-AFF	-.07	.19	-.01	-.05	.04	.39	.27	.24	-.19	-.22	-.27	1					
13. QSTR-DIS	.08	-.07	.16	.06	-.11	-.30	-.22	-.22	.32	.48	.42	-.42	1				
14. QSTR-BS	.06	-.08	.09	.17	-.04	-.25	-.28	-.20	.29	.39	.46	-.57	.65	1			
15. MATH-WC	.15	.12	.41	.24	.22	-.09	-.06	-.04	.03	.08	.05	.02	.17	.11	1		
16. MATH-REA	.26	.17	.28	.39	.27	-.33	-.33	-.19	-.02	.11	.12	-.04	.13	.13	.34	1	
17. MATH-NUM	.39	.19	.29	.28	.29	-.35	-.35	-.20	.05	.16	.12	-.07	.15	.16	.40	.62	1
M	28.07	10.33	12.8	16.72	125.22	25.17	19.71	11.64	77.57	76.04	75.68	6.56	18.01	25.61	6.17	16.43	19.86
SD	4.9	12.45	5.91	5.06	21.53	7.34	5.68	3.17	9.38	9.77	10.05	2.35	3.08	3.98	1.63	3.24	4.03

All coefficients > .15 are significant at .05 level. CAT: Cattell; PMA-S: Primary Mental Ability–Spatial; PMA-R: Primary Mental Ability–Reasoning; PMA-V: Primary Mental Ability–Verbal; VPTA: Visual Pattern Test–Active; MA-MLA: Mathematical Learning Anxiety; MA-MEA: Mathematical Evaluation Anxiety; MA-SGA: School Generalized Anxiety; MCS-A: Multidimensional Self-Concept Scale–Quality of interpersonal relationships; MCS-B: Multidimensional Self-Concept Scale–Emotional Competencies; MCS-C: Multidimensional Self-Concept Scale–Environment Capability; QSTR-AFF: Student-Teacher Relationship Questionnaire–Scale Affiliation with Teacher; QSTR-DIS: Student-Teacher Relationship Questionnaire–Scale Dissatisfaction with Teacher; QSTR-BS: Student-Teacher Relationship Questionnaire–Scale Bonds with School; MATH-WC: AC-MT–Written Calculation; MATH-REA: AC-MT–Mathematic reasoning; MATH-NUM: AC-MT–Number knowledge.

### Exploratory Factor Analysis (EFA)

We used a two-step modelling approach. In a first set of analyses, we used EFA to investigate the factorial structure of all our indicators. EFA was used to determine the adequate number of latent factor structures and to identify the number of factors underlying (conceptually and statistically) the set of items in each construct. The results from the EFA provided a clear estimation of the factor structure of the variables. We used Cattell’s scree plot test to identify the number of factors. Based on the results of the scree plot, five factors were clearly distinguishable. We named these factors according to the previous literature, specifically: general cognitive abilities, math anxiety, self-esteem, quality of student-teacher relationship, and mathematics achievement (Table 2). Confirmatory analyses were subsequently used to confirm the results of the EFA. Overall, about half of the variance (49.4%) was explained by these five factors.

**Table 2:** Factor loadings for the measurement model.

	Factor1	Factor2	Factor3	Factor4	Factor5
01. CAT	.68				
02. PMA-S	.50				
03. PMA-R	.57				
04. PMA-V	.32				
05. VPTA	.61				
06. MA-MLA		.92			
07. MA-MEA		.68			
08. MA-SGA		.51			
09. MCS-A			.76		
10. MCS -B			.84		
11. MCS -C			.77		
12. QSTR- AFF				-.54	
13. QSTR-DIS				.55	
14. QSTR-BS				.95	
15. MATH-WC					.41
16. MATH-REA					.78
17. MATH-NUM					.63

CAT: Cattell; PMA-S: Primary Mental Ability–Spatial; PMA-R: Primary Mental Ability–Reasoning; PMA-V: Primary Mental Ability–Verbal; VPTA: Visual Pattern Test–Active; MA-MLA: Mathematical Learning Anxiety; MA-MEA: Mathematical Evaluation Anxiety; MA-SGA: School Generalized Anxiety; MCS-A: Multidimensional Self-Concept Scale–Quality of interpersonal relationships; MCS-B: Multidimensional Self-Concept Scale–Emotional Competencies; MCS-C: Multidimensional Self-Concept Scale–Environment Capability; QSTR-AFF: Student-Teacher Relationship Questionnaire–Scale Affiliation with Teacher; QSTR-DIS: Student-Teacher Relationship Questionnaire–Scale Dissatisfaction with Teacher; QSTR-BS: Student-Teacher Relationship Questionnaire–Scale Bonds with School; MATH-WC: AC-MT–Written Calculation; MATH-REA: AC-MT–Mathematic reasoning; MATH-NUM: AC-MT–Number knowledge.

### Confirmatory Factor Analysis (CFA)

**Fig 1:**
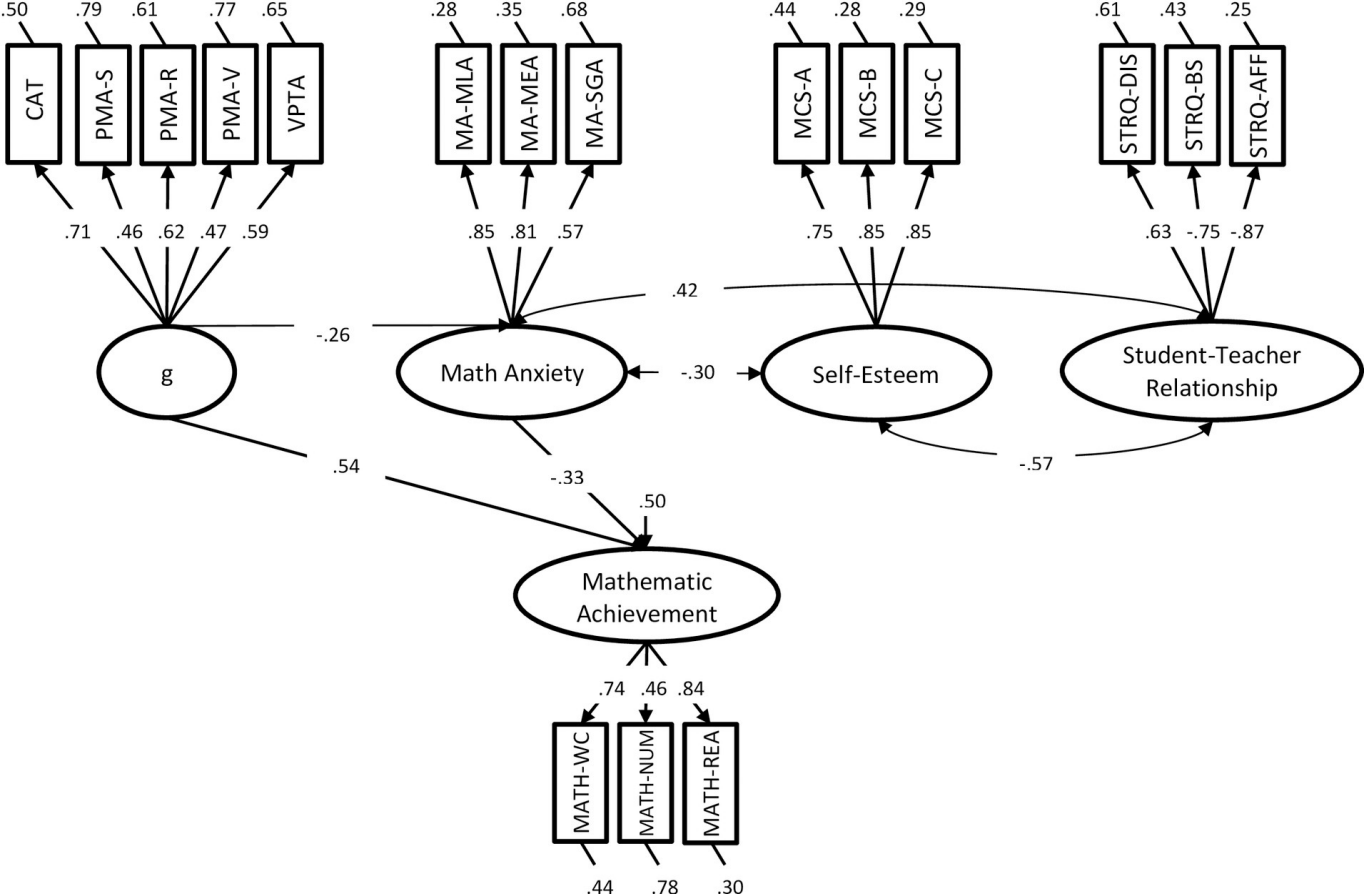
Model SEM-01.

Having established the factorial structure of our tasks, we attempted to confirm the factor structure using CFA (Table 3). In this model, the relationship between general cognitive abilities, math anxiety, self-esteem, the quality of the student-teacher relationship, and mathematics achievement were investigated. Based on the results of the EFA, we hypothesized the existence of five factors: general cognitive abilities (g), mathematics achievement (MATH), math anxiety (MA), self-esteem (SE), and the quality of the student-teacher relationship (QSTR). All loadings were statistically significant. The fit of this model (CFA-01) was adequate, *χ*^*2*^(109) = 178.71, *p* < .001, *RMSEA* = .06, *SRMR* = .07, *CFI* = .93, *NNFI* = .92, *AIC* = 18191 (Fig 1). This model was therefore used for subsequent analyses.

**Table 3:** Confirmatory factor analysis.

	GCA	MA	SE	QSTR	MATH
01. CAT	0.71				
02. PMA-S	0.46				
03. PMA-R	0.62				
04. PMA-V	0.48				
05. VPTA	0.59				
06. MA-MLA		0.85			
07. MA-MEA		0.81			
08. MA-SGA		0.57			
09. MCS-A			0.75		
10. MCS -B			0.85		
11. MCS -C			0.85		
12. QSTR- DIS				-0.63	
13. QSTR-BS				0.75	
14. QSTR-AFF				0.87	
15. MATH-WC					0.75
16. MATH-REA					0.84
17. MATH-NUM					0.47
GCA	1				
MA	-0.26*	1			
SE	0.07	-0.30*	1		
QSTR	0.07	-0.42*	0.57*	1	
MATH	0.62*	-0.48*	0.16^+^	0.21*	1

GCA: General Cognitive Abilities; MA: Math Anxiety; SE: Self-Esteem; QSTR: Quality of Student-Teacher Relationship; MATH: Mathematic Achievement; CAT: Cattell; PMA-S: Primary Mental Ability–Spatial; PMA-R: Primary Mental Ability–Reasoning; PMA-V: Primary Mental Ability–Verbal; VPTA: Visual Pattern Test–Active; MA-MLA: Mathematical Learning Anxiety; MA-MEA: Mathematical Evaluation Anxiety; MA-SGA: School Generalized Anxiety; MCS-A: Multidimensional Self-Concept Scale–Quality of interpersonal relationships; MCS-B: Multidimensional Self-Concept Scale–Emotional Competencies; MCS-C: Multidimensional Self-Concept Scale–Environment Capability; QSTR-AFF: Student-Teacher Relationship Questionnaire–Scale Affiliation with Teacher; QSTR-DIS: Student-Teacher Relationship Questionnaire–Scale Dissatisfaction with Teacher; QSTR-BS: Student-Teacher Relationship Questionnaire–Scale Bonds with School; MATH-WC: AC-MT–Written Calculation; MATH-REA: AC-MT–Mathematic reasoning; MATH-NUM: AC-MT–Number knowledge.

* p < .05, two tails

^+^ p < .05, one tail

### Structural Equation Modelling (SEM)

In Model SEM-01, all factors in CFA-01 were simultaneously predicting MATH (Fig 1). As for the correlations between exogenous factors, correlations that were not statistically significant in our measurement model (CFA-01) were not included. The fit of this model was adequate, *χ*^*2*^(109) = 178.71, *p* < .001, *RMSEA* = .06, *SRMR* = .07, *CFI* = .93, *NNFI* = .92, *AIC* = 18191. The average variance extracted (AVE) was .33, .60, .66, .67, .63 for g, MATH, MA, SE, and QSTR respectively. Overall, this model shows that only g and MATH are predicting unique portions of the variance. Such a finding might indicate that the relationship with MATH seems to be more articulated. Based on this finding and on the fact that MA, SE, and QSTR are highly intercorrelated we decided to run an additional model in which the relationship between SE and QSTR was mediated by MA. This model is also supported by the literature, which indicates that SE and QSTR are related to other constructs, such as MA [59, 122].

**Fig 2:**
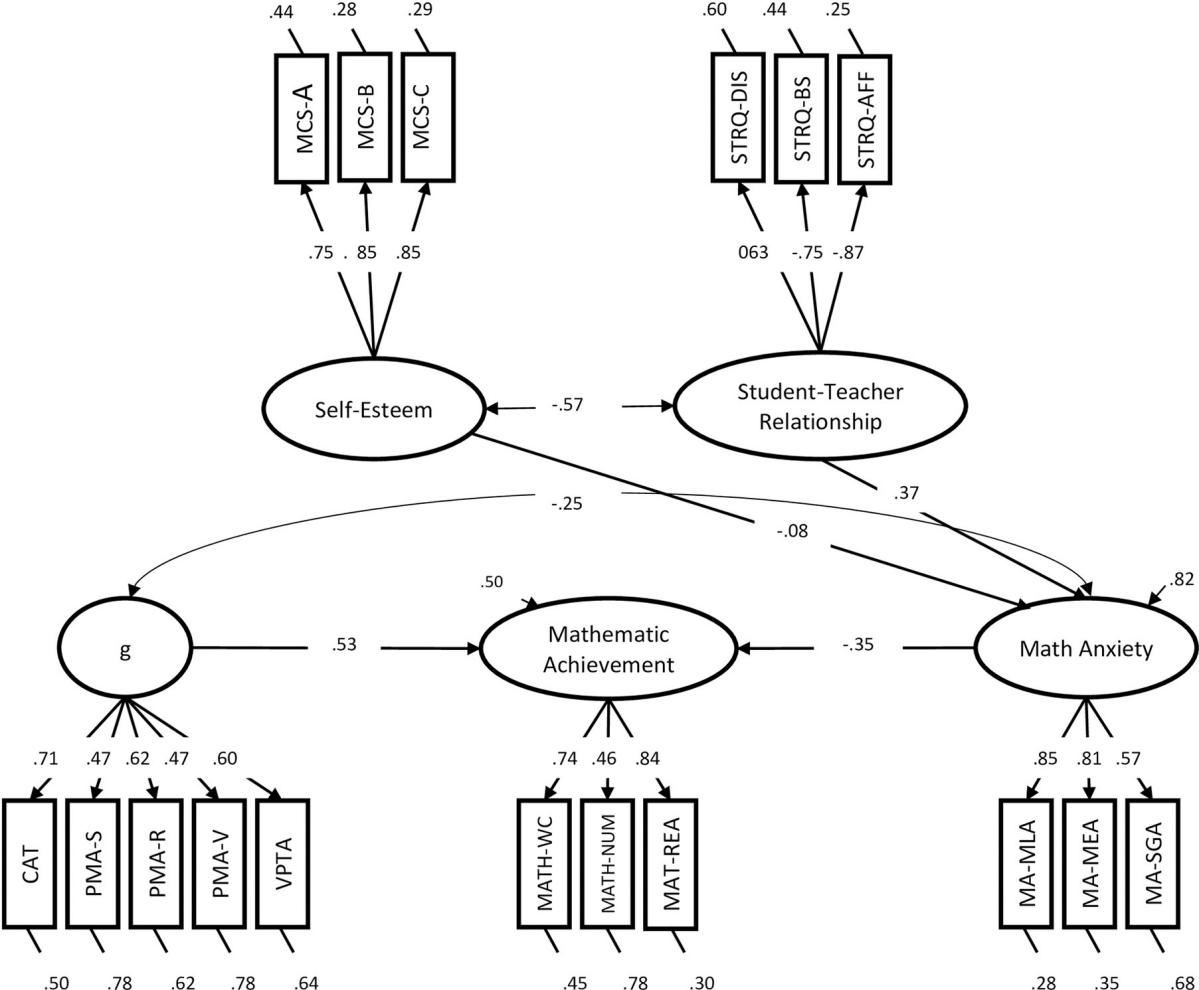
Model SEM-02.

In Model SEM-02, math anxiety mediated the effects of both self-esteem and QSTR on mathematics achievement (Fig 2). In the model, general cognitive ability and math anxiety were allowed to correlate. This model had a good fit, *χ*^*2*^(113) = 179.73, *p* < .001, *RMSEA* = .06, *SRMR* = .07, *CFI* = .94, *NNFI* = .92, *AIC* = 18184, and a lower AIC compared to model SEM-01 (*ΔAIC* = 7), meaning that this model should be retained. In this model, the proportion of variance explained by mathematics achievement was high (i.e., 50% of the variance). In this model, it was also possible to calculate indirect effects from QSTR and SE on MATH, with the mediation of MA. Notably, when tested, only the indirect effect of QSTR was statistically significant (*β* = -.13, *p* = .009), while the effect of SE was not (*β* = .03, *p* = .433). We decided to perform this analysis again using maximum likelihood estimation with robust (Huber-White, MLR) standard errors and a scaled test statistic that is (asymptotically) equal to the Yuan-Bentler test statistic. The fit of the model using this method, *χ*^*2*^(113) = 174.88, *p* < .001, *RMSEA* = .06, *SRMR(Bentler)* = .07, *CFI* = .94, *NNFI* = .93, was very similar to the fit calculated with the traditional ML method. This method also allowed us to adjust and correct SEs, providing more reliable estimates; results using this method are very similar to those obtained with the traditional ML and are not reported due to redundancy.

### Additional analyses

**Fig 3:**
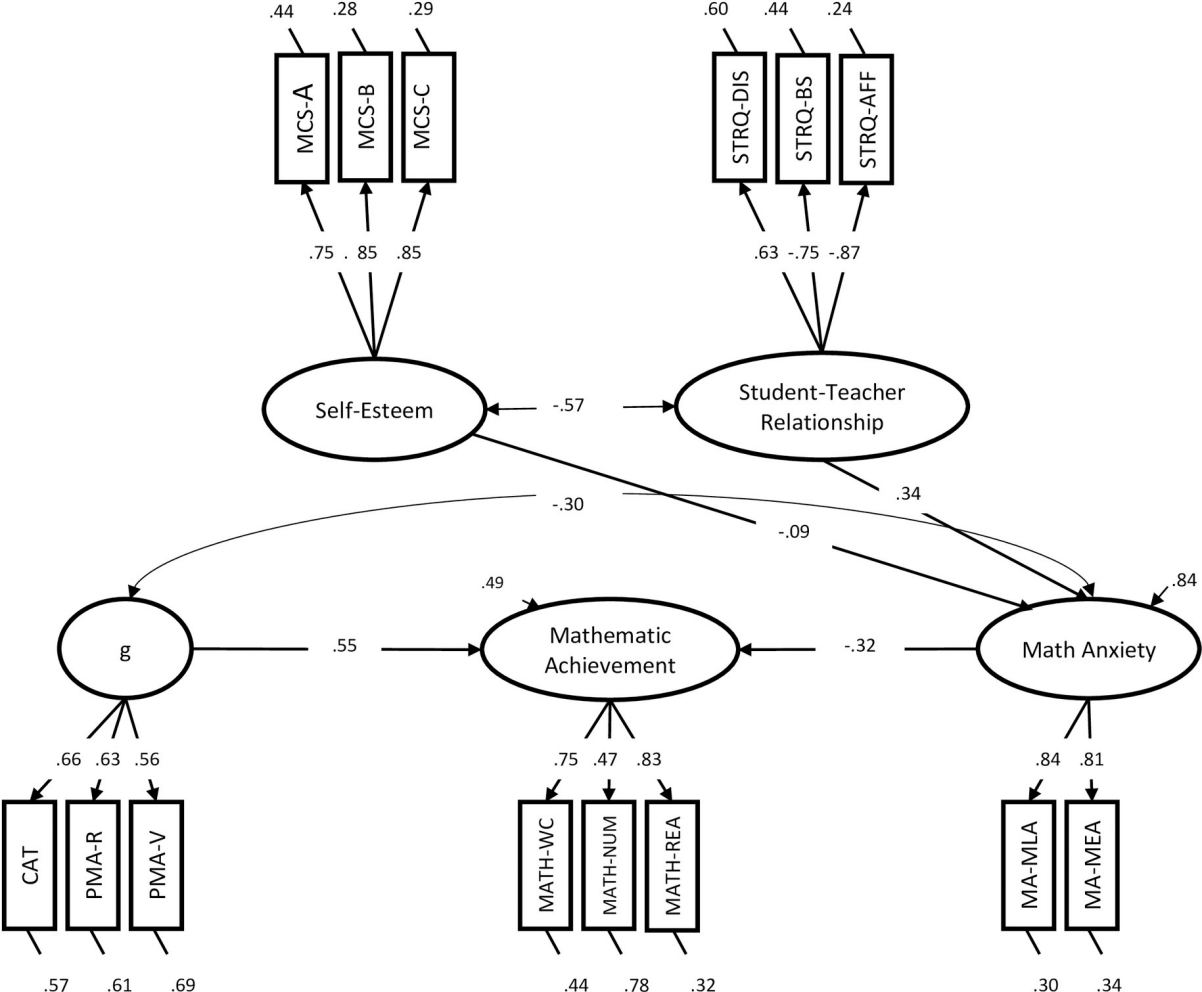
Model SEM-03.

General cognitive ability encompasses a series of important constructs, such as working memory (WM). Working memory is a highly related but separable construct from intelligence ([123] for a review). In fact, some of the tasks included in this paper as measures of general cognitive abilities could be defined as WM tasks. These tasks include: the VPTA, which requires the subject to maintain and manipulate information for a short period of time and, to a lesser extent, the PMA-S, which requires the subject to mentally hold and mentally manipulate visual objects. For this reason, we decided to run model SEM-02 again without including these tasks in the model. In a similar vein, School Generalized Anxiety, which was included in the MA factor, is probably measuring some anxiety components related to general anxiety rather than being specific to math anxiety. For all these reasons, we decided to perform a new model SEM-03, very similar to model SEM-02, but in which some measures (i.e., VPTA, PMA-S and School Generalized Anxiety) were excluded. This gave us the opportunity to have measures of General Cognitive Abilities less dependent on WM, and measures of Math anxiety less influenced by other factors such as general anxiety. The fit of model SEM-03, χ2(71) = 118.12, p < .001, RMSEA = .06, SRMR = .06, CFI = .95, NNFI = .93, AIC = 14324, was very similar to, and slightly better than, model SEM-02. The model’s parameters were very similar to those in model SEM-02, and all the patterns that were statistically significant in model SEM-02 remained statistically significant in model SEM-03. Model SEM-03 is presented in Fig 3.

## Discussion

The current study was devoted to investigate the relationship between general cognitive abilities, math anxiety, self-esteem, the quality of the student-teacher relationship, and their joint effects on mathematics achievement. The influence of general cognitive abilities on mathematics achievement has been widely explored in the literature [22]. The role of math anxiety and self-esteem, among the non-cognitive factors, has also been considered by several studies [20]. In addition, there is an increasing body of research suggesting that the quality of the student-teacher relationship plays an important role in mathematics achievement [69, 124, 125]. However, with the present study we tried to overcome some of the pitfalls of previous studies; indeed, although these constructs have been widely explored for kindergarten [126] and primary school [81], further research seems necessary for middle school [127]. Additionally, to our knowledge, no study has explored the joint effects of these factors for adolescents transitioning to middle school.

As expected, mathematics achievement and general cognitive abilities were very closely linked yet distinguishable from each other, as they only shared about half of the variance [20, 128]. Several studies corroborate these results, identifying general cognitive abilities as a relevant predictor of mathematics achievement (e.g. [128–132]). In stages of transition in schooling, such as the transition from elementary to middle school, general cognitive abilities play a more relevant role for mathematics learning, helping students to acquire the new skills and abilities required to learn new academic subjects and topics [132]. Notably, although we considered the influence of general cognitive ability on mathematics achievement, general cognitive ability explains only a portion of the mathematical variance, meaning that other variables also seem to play a role in mathematics achievement. For example, non-cognitive factors (e.g., math anxiety and self-esteem, among several others) tend to be consistently associated with mathematics achievement. With this respect, in line with previous evidence, we found a strong negative relationship between math anxiety and mathematics achievement [133–138]. The widespread findings of a negative relationship between math anxiety and performance could be interpreted in the light of studies that showed how, in this age group, students tend to implement avoidance behaviors towards mathematics [35]. This avoidance might cause students to fall even further behind in their math understanding; therefore, our findings suggest the importance of addressing math anxiety in order to improve math performance in this particular age group [139]. Efforts to curb math anxiety need to include ways to treat it in those who are already experiencing it, as well as ways to prevent it in the first place; this is why recent literature tends to focus on other factors, such as self-esteem [59, 140].

Our results showed, also, that the effects of self-esteem and the quality of the student-teacher relationship on mathematics achievement was mediated by math anxiety. As to the role played by self-esteem, the nonsignificant indirect effects on mathematics achievement were in continuity with other studies [141, 142]. This finding should be interpreted in light of the joint effects of the other variables of the model. In fact, analyzing the correlation table the relation between self-esteem and math anxiety was moderate, while the association between self-esteem and mathematics achievement was small. This effect could be interpreted in the light of the association between self-esteem and the quality of student-teacher relationship. Indeed, the correlation table showed a strong association between these measures. Therefore, the quality of student-teacher relationship explained a greater variance of the model, also absorbing the effects of self-esteem. Our results highlighted the need for further studies to disentangle the complex nature of the construct and its relation with other measures. This finding seems to indicate that other non-cognitive factors are involved, and, among others, the student-teacher relationship seems to be a prominent factor.

The quality of the student-teacher relationship is associated with mathematics achievement, through the mediation of math anxiety, thus a good quality of the relationships with the teachers dampens math anxiety, leading to effective math performances. There is growing research suggesting that student-teacher relationships may be important for mathematics achievement [84, 143]. However, to the best of our knowledge, this is the first study supporting such indirect pathway. It can be argued that a teacher’s emphasis on the expression of warmth, responsiveness, trust, and accessibility could support students in regulating appropriately their math anxiety, leading to a positive impact on mathematics performance. In accordance with predictions from the attachment framework, teachers act as secure bases for their students, by increasing the child’s confidence in exploring new learning materials and regulating anxiety when facing novelties, thus contributing to reducing math anxiety and positively predicting math achievement [62]. Our results thus confirm that a positive student-teacher relationship could influence mathematics achievement [65].

This study may have important implications for teacher training and professional development. For a start, the teacher’s role in promoting an emotionally supportive context and creating warm, respectful relationships with all students by empowering their socio-emotional skills should not be overlooked in favor of concerns about curriculum and direct instruction. Our findings suggest the importance of emphasizing the formation and the maintenance of close relationships between students and teachers for younger, as well as older, children (e.g., those included in the current study). This study provide evidence that teachers who build emotionally supportive relationships with their students may have an important impact on their mathematics achievement. This type of closeness may be particularly important for improving mathematics achievement, which for children requires continued engagement with the academic material and continued practice of those competencies outside of the school context [144].

Though it contains some insightful findings, the present study also has some limitations that should be addressed by future studies. Firstly, the cross-sectional design does not allow to draw conclusions about causal relations and ways of causation. Longitudinal studies are needed to analyze how the correlations between all the variables shift over time, and especially how they influence children’s performance in general and mathematical achievement specifically. Future studies should consider how the form of the relationship might vary across time. For example, it might be that student-teacher relationships are most important in the 6^th^ grade but become less important in the transition to higher levels of education. In fact, this age group was chosen based on the observation that early genetic influences on cognition are amplified over time, and innovative genetic influences seem to arise with time [145]. However, these findings are currently under debate, and more research is needed to understand these changes over time. Future studies should also consider methods for examining the causal effects of student-teacher relationships and the impact of closeness and conflict on other subjects of the academic curriculum (e.g., reading literacy). Notably, in the present paper, half of the mathematics achievement variance was explained by the factors included in the present report, meaning other factors may also play a significant role in mathematics performance. For example, resilience seems to be positively associated with mathematics performance but was not included in the present report. Furthermore, the overall school climate also seems to play a significant role in academic performance [146]. It is also noteworthy that a convenience sample was employed in the current research and that our agreement with the schools only allowed us to test each child for a limited amount of time. Therefore, these results should be replicated with a larger sample of the population and using a broader assessment, e.g., including more tasks, and tapping executive functions, working memory, and other important aspects usually subsumed under the g-factor. Finally, using CFA and SEM does not permit the testing of the complex interlinks that might exist between factors included in the current research; therefore, these results must be considered only preliminary.

Notwithstanding these limitations, this is the first study that testing the joint influence of general cognitive abilities, math anxiety, self-esteem and the quality of student-teacher relationship on mathematics achievement. Furthermore, we decided to focus on objective and standardized measures to assess mathematics achievement, and not on an evaluation provided by the teachers (e.g., grade point average) that could be influenced by personality and other non-cognitive factors. Lastly, we decided to use a latent modelling approach, which gave us the possibility of modeling complex dependencies and structural models and of reducing the measurement errors typical in more traditional approaches. To summarize, the present study has shown that several factors, both cognitive and non-cognitive, play an important role in mathematics achievement; furthermore, the results seem to indicate that the quality of the student-teacher relationship may have an effect on mathematics achievement. These findings have a number of strengths that could help to build upon the research on student-teacher relationships and mathematics achievement.

## References

[1] Higbee JL, Thomas PV. Affective and cognitive factors related to mathematics achievement. J Develop Educ. 1999;23(1):8.

[2] Lu L, Weber HS, Spinath FM, Shi J. Predicting school achievement from cognitive and non-cognitive variables in a Chinese sample of elementary school children. Intelligence. 2011;39(2–3):130–140.

[3] Fonteyne L, Duyck W, De Fruyt F. Program-specific prediction of academic achievement on the basis of cognitive and non-cognitive factors. Learn Individ Differ. 2017;56,34–48.

[4] Devine A, Hill F, Carey E, Szűcs D. Cognitive and emotional math problems largely dissociate: Prevalence of developmental dyscalculia and mathematics anxiety. J Educ Psychol. 2018;110(3):431–444.

[5] Lee K, Ning F, Goh HC. Interaction between cognitive and non-cognitive factors: The influences of academic goal orientation and working memory on mathematical performance. Educ Psychol. 2014;34(1):73–91.

[6] Passolunghi MC, Cargnelutti E, Pellizzoni S. The relation between cognitive and emotional factors and arithmetic problem-solving. Educ Stu Mathem, 2019;100(3): 271–290.

[7] Baten E, Desoete A. Mathematical (Dis) abilities within the opportunity propensity model: the choice of math test matters. Front Psychol. 2018;9:667 10.3389/fpsyg.2018.00667 29867645 PMC5952253

[8] Ramirez G, Shaw ST, Maloney EA. Math anxiety: Past research, promising interventions, and a new interpretation framework. Educ Psychol. 2018;53(3):145–164.

[9] Chang H, Beilock SL. The math anxiety-math performance link and its relation to individual and environmental factors: a review of current behavioral and psychophysiological research. Curr Opin Behav Sc. 2016;10:33–38.

[10] Caputi M, Lecce S, Pagnin A. The role of mother–child and teacher–child relationship on academic achievement. Eur J Dev Psychol. 2017;14(2):141–158.

[11] Zee M, de Bree E. Students’ self-regulation and achievement in basic reading and math skills: the role of student–teacher relationships in middle childhood. Eur J Dev Psychol. 2016;10:1–16.

[12] Gottfredson LS. Mainstream science on intelligence: An editorial with 52 signatories, history, and bibliography. Intelligence. 1997;24:79–132.

[13] Kaufman SB. Beyond general intelligence: the dual-process theory of human intelligence Yale University 2009.

[14] Kaufman SB, Reynolds MR, Liu X, Kaufman AS, McGrew KS. Are cognitive g and academic achievement g one and the same g? An exploration on the Woodcock–Johnson and Kaufman tests. Intelligence. 2012;40(2):123–138.

[15] McGrew KS. CHC theory and the human cognitive abilities project: Standing on the shoulders of the giants of psychometric intelligence research. Intelligence. 2009;37:1–10.

[16] Horn JL, Cattell RB. Refinement and test of the theory of fluid and crystallized general intelligences. J Educ Psychol. 1966;57(5):253 10.1037/h0023816 5918295

[17] Horn JL, Hofer SM. Major abilities and development in the adult period In Sternberg R. J. & Berg C. A. (Fais.), Intell Dev. 1992;44–99. New York: Cambridge University Press.

[18] Horn JL, Noll J. Human cognitive capabilities: Gf–Gc theory In Flanagan D. P., Genshaft J. L., & Harrison P. L. (Eds.), Contemporary intellectual assessment: Theories, tests and issues. 1997;53−91. New York: Guilford.

[19] McGrew KS. The Cattell–Horn–Carroll theory of cognitive abilities In Flanagan D. P., & Harrison P. L. (Eds.), Contemporary intellectual assessment: Theories, tests, and issues. 2005;136−181.2nd ed. New York: Guilford Press.

[20] Giofrè D, Borella E, Mammarella IC. The relationship between intelligence, working memory, academic self-esteem, and academic achievement. J Cogn Psychol. 2017 4;5911:1–17.

[21] Soares DL, Lemos GC, Primi R, Almeida LS. The relationship between intelligence and academic achievement throughout middle school: The role of students’ prior academic performance. Learn IndividDiffer. 2015 7;41:73–8.

[22] Bizzaro M, Giofrè D, Girelli L, Cornoldi C. Arithmetic, working memory, and visuospatial imagery abilities in children with poor geometric learning. Learn Individ Differ. 2018 2;62:79–88.

[23] Hunt E. Human Intelligence. New York, NY: Cambridge University Press; 2011 pp. 1–528.

[24] Fuchs LS, Fuchs D, Compton DL, Hamlett CL, Wang AY. Is word-problem solving a form of text comprehension?. Scientific Studies of Reading. 2015;19(3), 204–223. 10.1080/10888438.2015.1005745 25866461 PMC4391820

[25] Swanson HL. Working memory in learning disability subgroups. J Exp Child Psychol. 1993;56: 87–114. 10.1006/jecp.1993.1027 8366327

[26] Geary DC. The Origin of Mind: Evolution of Brain, Cognition, and General Intelligence. In Genes Brain and Behavior. 2006;163(9).

[27] Halberda J, Mazzocco MM, Feigenson L. Individual differences in non-verbal number acuity correlate with maths achievement. Nature. 2008;455(7213), 665 10.1038/nature07246 18776888

[28] Peng P, Wang C, Wang T, Lin X. A Meta-analysis on the Relation between Fluid Intelligence and Reading/Mathematics: Effects of Tasks, Age, and Social Economics Status. 10.1037/bul0000182 30652909

[29] Psychol Bull. 2019;145(2),189–236. 10.1037/bul0000182 30652909

[30] Carey E, Hill F, Devine A, Szücs D. The chicken or the egg? The direction of the relationship between mathematics anxiety and mathematics performance. Front Psychol. 2016;6:1987 10.3389/fpsyg.2015.01987 26779093 PMC4703847

[31] Dowker A, Sarkar A, Looi CY. Mathematics anxiety: what have we learned in 60 years? Front Psychol. 2016. 10.3389/fpsyg.2016.00508 PMC4842756 27199789

[32] Ramirez G, Shaw ST, Maloney EA. Math anxiety: Past research, promising interventions, and a new interpretation framework. Educ Psychol. 2018;53(3), 145–164.

[33] Ashcraft MH, Ridley KS. Math anxiety and its cognitive consequences. Am Psychol Soc. 2002.

[34] Ashcraft MH, Ridley KS. Math anxiety and its cognitive consequences: A tutorial review In: Campbell JID, editor. Handbook of mathematical cognition. New York, NY: Psychology Press; 2005 pp. 315–327.

[35] Ma X, Xu J. The causal ordering of mathematics anxiety and mathematics achievement: a longitudinal panel analysis. J Adolesc. 2004;27(2):165–79. 10.1016/j.adolescence.2003.11.003 15023516

[36] Maloney EA. Math anxiety: Causes, consequences, and remediation. In Wentzel K. R. & Miele D. B. (Eds.), Handbook of Motivation at School. 2016; (2nd ed., 408–423). New York, NY: Routledge.

[37] Meece JL, Wigfield A, Eccles JS. Predictors of Math Anxiety and Its Influence on Young Adolescents’ Course Enrollment Intentions and Performance in Mathematics. J Educ Psychol.1990; 82(1), 60.

[38] Wigfield A, Meece JL. Math anxiety in elementary and secondary school students. J Educ Psychol. 1988;80(2), 210.

[39] Diseth Å, Meland E, Breidablik HJ. Self-beliefs among students: Grade level and gender differences in self-esteem, self-efficacy and implicit theories of intelligence. Learn Individ Differ. 2014;35:1–8.

[40] Hansford BC, Hattie JA. The relationship between self and achievement/performance measures. Rev Educ Res. 1982;52(1):123–142.

[41] Lee J. Universals and specifics of math self-esteem, math self-efficacy, and math anxiety across 41 PISA 2003 participating countries. Learn Individ Differ. 2009;19(3):355–65.

[42] Marsh HW, Hau KT. Big-Fish-Little-Pond effect on academic self-esteem: A cross-cultural (26-country) test of the negative effects of academically selective schools. Am Psychol. 2003;58(5):364 10.1037/0003-066x.58.5.364 12971085

[43] Marsh HW, Hau KT. Explaining paradoxical relations between academic self-esteems and achievements: Cross-cultural generalizability of the internal/external frame of reference predictions across 26 countries. J Educ Psychol. 2004;96(1):56.

[44] Orth U., & Robins R. W. (2014). The development of self-esteem. Curr Dir Psychol Sci, 23(5), 381–387.

[45] Cvencek D, Fryberg SA, Covarrubias R, Meltzoff AN. Self‐concepts, self‐esteem, and academic achievement of minority and majority north American elementary school children. Child Dev. 2018;89(4), 1099–1109. 10.1111/cdev.12802 28386954

[46] Harter S. Self-perception profile for adolescents: Manual and questionnaires 2012 Denver, CO: Univeristy of Denver, Department of Psychology.

[47] Baumeister RF, Campbell JD, Krueger JI, Vohs KD. Does high self-esteem cause better performance, interpersonal success, happiness, or healthier lifestyles?. 10.1111/1529-1006.01431 26151640

[48] Psychol Sci Public Interest. 2003;4(1):1–44. 10.1111/1529-1006.01431 26151640

[49] Aryana M. Relationship between self-esteem and academic achievement amongst pre-university students. J Appl Sci. 2010;10(20):2474–2477.

[50] Colquhoun LK, Bourne PA. Self-Esteem and Academic Performance of 4^(th) Graders in two Elementary Schools in Kingston and St. Andrew, Jamaica. Asian Journal of Business Management. 2012;4(1):36–57.

[51] Das PPP, Pattanaik P. Self-Esteem, Locus of Control and Academic Achievement among Adolescents. International Journal of Scientific Research in Recent Sciences. 2013;1(01):1–5.

[52] Olanrewaju MK, Joseph OB. Academic Efficacy and Self-Esteem as Predictors of Academic Achievement among School Going Adolescents in Itesiwaju Local Government Area of Oyo State, Nigeria. J Educ Prac. 2014;22(5), 169–175.

[53] Lane J, Lane AM, Kyprianou A. Self-efficacy, self-esteem and their impact on academic performance. Soc Behav Pers: an international journal. 2004;32(3):247–256.

[54] Alves-Martins M, Peixoto F, Gouveia-Pereira M, Amaral V, Pedro I. Self-esteem and Academic Achievement Among Adolescents. Educ Psychol: An International Journal of Experimental Educational Psychology. 2010;22(1):51–62.

[55] Trautwein U, Lüdtke O, Köller O, Baumert J. Self-esteem, academic self-concept, and achievement: How the learning environment moderates the dynamics of self-concept. J Pers Soc Psychol. 2006;90(2):334 10.1037/0022-3514.90.2.334 16536654

[56] Akin A, Kurbanoglu IN. The relationships between math anxiety, math attitudes, and self-efficacy: A structural equation model. Stud Psychol. 2011;53(3):263.

[57] Bourquin SD. The relationship among math anxiety, math self-efficacy, gender, and math achievement among college students at an open admissions commuter institution. Dissertation Abstracts International, Section A: Humanities and Social Sciences. 1999;60(3-A):0679.

[58] Hackett G. The role of mathematics self-efficacy in the choice of math-related majors of college women and men: A path analysis. J Couns Psychol. 1985;32, 47–56.

[59] Pajares F, Graham L. Self-efficacy, motivation constructs, and mathematics performance of entering middle school students. Contemp Educ Psychol. 1999;24:124–139. 10.1006/ceps.1998.0991 10072312

[60] Ahmed W, Minnaert A, Kuyper H, van der Werf G. Reciprocal relationships between math self-concept and math anxiety. Learn Individ Differ. 2012;22(3):385–389.

[61] Xie F, Xin Z, Chen X, Zhang L. Gender Difference of Chinese High School Students’ Math Anxiety: The Effects of Self-Esteem, Test Anxiety and General Anxiety. Sex Roles. 2019;81(3–4):235–244.

[62] Cadima J, Doumen S, Verschueren K, Buyse E. Child engagement in the transition to school: Contributions of self-regulation, teacher-child relationships and classroom climate. Early Child Res Q. 2015;32:1–12

[63] Midgley C, Feldlaufer H, Eccles JS. Change in Teacher Efficacy and Student Self- and Task-Related Beliefs in Mathematics During the Transition to Junior High School. J Educ Psychol. 1989;81(2):247.

[64] Sabol TJ, Pianta RC. Recent trends in research on teacher–child relationships. Attach Hum Dev. 2012;14(3). 10.1080/14616734.2012.672262 22537521

[65] Bowlby J. Attachment, Vol. 1 of Attachment and loss. London: Hogarth Press;1969.

[66] Pianta RC. Enhancing relationships between children and teachers Washington: American Psychological Association 1999

[67] Roorda DL, Koomen HMY, Spilt JL, Oort FJ. The Influence of affective teacher-student relationships on students’ school engagement and achievement: A meta-analytic approach. Rev Educ Res. 2011;81(4):493–529.

[68] Hamre BK, Pianta RC. Can instructional and emotional support in the first‐grade classroom make a difference for children at risk of school failure?. Child Dev. 2005;76(5):949–967. 10.1111/j.1467-8624.2005.00889.x 16149994

[69] Fast LA, Lewis JL, Bryant MJ, Bocian KA, Cardullo RA, Rettig M, et al Does math self-efficacy mediate the effect of the perceived classroom environment on standardized math test performance?. J Educ Psychol. 2010;102(3):729.

[70] Sakiz G, Pape SJ, Hoy AW. Does perceived teacher affective support matter for middle school students in mathematics classrooms? J Sch Psychol. 2012; 50(2):235–255. 10.1016/j.jsp.2011.10.005 22386122

[71] Tosto MG, Asbury K, Mazzocco MM, Petrill SA, Kovas Y. From classroom environment to mathematics achievement: The mediating role of self-perceived ability and subject interest. Learn Individ Differ. 2016;50:260–269. 10.1016/j.lindif.2016.07.009 27766018 PMC5063534

[72] Wentzel KR, Battle A, Russell SL, Looney LB. Social supports from teachers and peers as predictors of academic and social motivation. Contemp Educ Psychol. 2010;35(3):193–202.

[73] Furrer C, Skinner E. Sense of relatedness as a factor in children’s academic engagement and performance. J Educ Psychol. 2003;95(1):148.

[74] Marchand G, Skinner EA. Motivational dynamics of children’s academic help-seeking and concealment. J Educ Psychol. 2007; 99(1):65.

[75] Niehaus K, Rudasill KM, Rakes CR. A longitudinal study of school connectedness and academic outcomes across sixth grade. J Sch Psychol. 2012;50(4):443–460. 10.1016/j.jsp.2012.03.002 22710015

[76] Pianta RC, Hamre BK. Classroom processes and positive youth development: Conceptualizing, measuring, and improving the capacity of interactions between teachers and students. New Dir Youth Dev. 2009;121:33–46. 10.1002/yd.295 19358233

[77] Martin DP, Rimm-Kaufman SE. Do student self-efficacy and teacher-student interaction quality contribute to emotional and social engagement in fifth grade math? J Sch Psychol. 2015;53(5):359–373. 10.1016/j.jsp.2015.07.001 26407834

[78] Rudasill KM, Reio TG, Stipanovic N, Taylor JE. A longitudinal study of student-teacher relationship quality, difficult temperament, and risky behavior from childhood to early adolescence. J Sch Psychol. 2010; 48(5):389–412. 10.1016/j.jsp.2010.05.001 20728689

[79] Hughes J, Kwok OM. Influence of student-teacher and parent-teacher relationships on lower achieving readers’ engagement and achievement in the primary grades. J Educ Psychol. 2007;99(1):39 10.1037/0022-0663.99.1.39 18084625 PMC2140005

[80] Murray C, Murray KM. Child level correlates of teacher–student relationships: An examination of demographic characteristics, academic orientations, and behavioral orientations. Psychol Sch. 2004;41(7):751–762.

[81] Hamre BK, Pianta RC. Can instructional and emotional support in the first‐grade classroom make a difference for children at risk of school failure?. Child Dev. 2005;76(5):949–967. 10.1111/j.1467-8624.2005.00889.x 16149994

[82] Bryce D, Whitebread D. The development of metacognitive skills: Evidence from observational analysis of young children’s behavior during problem-solving. Metacogn Learn. 2012;7(3):197–217.

[83] McKinnon RD, Blair C. Bidirectional relations among executive function, teacher–child relationships, and early reading and math achievement: A cross-lagged panel analysis. Early Child Res Q. 2019;46:152–165.

[84] Blair C, Razza RP. Relating effortful control, executive function, and false belief understanding to emerging math and literacy ability in kindergarten. Child Dev. 2007;78(2):647–663. 10.1111/j.1467-8624.2007.01019.x 17381795

[85] Hamre B, Hatfield B, Pianta R, Jamil F. Evidence for general and domain‐specific elements of teacher–child interactions: Associations with preschool children’s development. Child Dev. 2014;85(3):1257–1274. 10.1111/cdev.12184 24255933

[86] Crosnoe R, Leventhal T, Wirth RJ, Pierce KM, Pianta RC. Family socioeconomic status and consistent environmental stimulation in early childhood. Child Dev. 2010;81(3):972–987. 10.1111/j.1467-8624.2010.01446.x 20573117 PMC2892811

[87] Fuchs L, Fuchs D, Prentice K, Burch M, Hamlett CL, Owen R, et al Enhancing third-grade student’mathematical problem solving with self-regulated learning strategies. J Educ Psychol. 2003;95(2):306.

[88] Rucinski CL, Brown JL, Downer JT. Teacher–child relationships, classroom climate, and children’s social-emotional and academic development. J Educ Psychol. 2018; 110(7):992.

[89] Passolunghi MC, Caviola S, De Agostini R, Perin C, Mammarella IC. Mathematics anxiety, working memory, and mathematics performance in secondary-school children. Front Psychol. 2016;7:42 10.3389/fpsyg.2016.00042 26869951 PMC4735424

[90] Wigfield A, Eccles JS, Mac Iver D, Reuman DA, Midgley C. Transitions During Early Adolescence: Changes in Children’s Domain-Specific Self-Perceptions and General Self-Esteem Across the Transition to Junior High School. Dev Psychol. 1991; 27(4):552.

[91] Trezise K, Reeve RA. Worry and working memory influence each other iteratively over time. Cogn Emot. 2015;3:1–16. 10.1080/02699931.2014.1002755 25648296

[92] Trezise K, Reeve RA. Working memory, worry, and algebraic ability. J Exp Child Psychol. 2014;121:120–136. 10.1016/j.jecp.2013.12.001 24487226

[93] Pekrun R, Elliot AJ, Maier MA. Achievement goals and discrete achievement emotions: A theoretical model and prospective test. J Educ Psychol. 2006;98(3):583.

[94] Pekrun R, Elliot AJ, Maier MA. Achievement goals and achievement emotions: Testing a model of their joint relations with academic performance. J Educ Psychol. 2009;101(1):115.

[95] Cattell RB, Cattell AKS. Misurare l’intelligenza con i test “Culture Fair.” Firenze: Organizzazioni Speciali;1981.

[96] Gregory RJ. Psychological testing: History, principles, and applications Needham Heights, MA: Allyn & Bacon 2004.

[97] Thurstone LL, Thurstone TG. Primary Mental Abilities: Examiner’s Manual. For Grades K-1. Sci Res Assoc. 1963.

[98] Mammarella IC, Borella E, Pastore M, Pazzaglia F. The structure of visuospatial memory in adulthood. Learn Individ Differ. 2013; 25:99–110.

[99] Mammarella IC, Cornoldi C, Pazzaglia F, Toso C, Grimoldi M, Vio C. Evidence for a double dissociation between spatial-simultaneous and spatial-sequential working memory in visuospatial (nonverbal) learning disabled children. Brain Cogn. 2006;62(1):58–67. 10.1016/j.bandc.2006.03.007 16750287

[100] Della Sala S, Gray C, Baddeley A, Wilson L. The Visual Patterns Test: A new test of short-term visual recall. Suffolk: Thames Valley Test Company 1997.

[101] Caponi B, Cornoldi C, Falco G, Focchiatti R, Lucangeli D. MeMa. Valutare la metacognizione, gli atteggiamenti negativi e l’ansia in matematica. Trento:Erikson; 2012.

[102] Bracken BA. Multidimensional self esteem scale Texas:Pro-ed;1992.

[103] Renaud-Dubé A, Guay F, Talbot D, Taylor G, Koestner R. The relations between implicit intelligence beliefs, autonomous academic motivation, and school persistence intentions: a mediation model. Soc Psychol Edu. 2015;18(2):255–272.

[104] Robins RW, Pals JL. Implicit self-theories in the academic domain: Implications for goal orientation, attributions, affect, and self-esteem change. Self Identity. 2002;1(4):313–336.

[105] Arens AK, Yeung AS, Craven RG, Hasselhorn M. The twofold multidimensionality of academic self-concept: Domain specificity and separation between competence and affect components. J Educ Psychol. 2011;103:970–981.

[106] Arens AK, Hasselhorn M. Differentiation of competence and affect self-perceptions in elementary school students: extending empirical evidence. European journal of psychology of education. 2015;30(4):405–419.

[107] Arens AK, Marsh HW, Craven RG, Yeung AS, Randhawa E, Hasselhorn M. Math self-concept in preschool children: Structure, achievement relations, and generalizability across gender. Early Child Res Q. 2016;36:391–403.

[108] Burns RA, Crisp DA, Burns RB. Competence and affect dimensions of self-concept among higher education students: a factorial validation study of an academic subject-specific self-concept. European Journal of Psychology of Education. 2018;33(4):649–663.

[109] Marsh HW, Dowson M, Pietsch J, Walker R. Why multicollinearity matters: a reexamination of relations between self-efficacy, self-concept, and achievement. J Educ Psychol. 2004;96(3):518.

[110] Pietsch J, Walker R, Chapman E. The relationship among self-concept, self-efficacy, and performance in mathematics during secondary school. J Educ Psychol. 2003;95(3):589.

[111] Pinxten M, Marsh HW, De Fraine B, Van Den Noortgate W, Van Damme J (2014). Enjoying mathematics or feeling competent in mathematics? Reciprocal effects on mathematics achievement and perceived math effort expenditure. Br J Educ Psychol. 2014;84(1):152–174. 24547759 10.1111/bjep.12028

[112] Yang L, Arens AK, Watkins DA. Testing the twofold multidimensionality of academic self-concept: a study with Chinese vocational students. Educ Psychol. 2016;36(9):1651–1669.

[113] Tonci E, De Domini P, Tomada G. How to evaluate the student-teacher relationship: Italian adaptation of the Student-Teacher Relationship Questionnaire by Murray and Greenberg in a integrate perspective. Italian journal of psychology. 2012;39(3):667–96.

[114] Murray C, Greenberg MT. Relationships with teachers and bonds with school: Social emotional adjustment correlates for children with and without disabilities. Psychology in the School. 2001;38(1):25–41.

[115] Bowlby J. Attachment and loss: Retrospect and prospect. Am J Orthopsychiatry. 1982;52(4):664 10.1111/j.1939-0025.1982.tb01456.x 7148988

[116] Armsden GC, Greenberg MT. The inventory of parent and peer attachment: individual differences and their relationship to psychological well-being in adolescence. J Youth Adolesc. 1987;16(5):427–54. 10.1007/BF02202939 24277469

[117] Hawkins JD, Catalano R, Morrison D, O’Donnell J, Abbott R, Day L. The Seattle Social Development Project: Effects of the first four years on protective factors and problem behaviors In An earlier version of this chapter was presented at the Society for Research in Child Development, Kansas City, Missouri, Apr 1989. Guilford Press 1992;139–61.

[118] Cornoldi C, Cazzola C. AC-MT 11–14: Test per la valutazione delle difficoltà di calcolo. Trento:Erikson;2004.

[119] Team RC. R: A language and environment for statistical computing. 2013.

[120] Revelle WR. Psych: Procedures for personality and psychological research. 2017.

[121] Rosseel Y. Lavaan: An R package for structural equation modeling and more. Version 0.5–12 (BETA). J Stat Softw. 2012;48(2):1–36.

[122] Hu LT, Bentler PM. Cutoff criteria for fit indexes in covariance structure analysis: Conventional criteria versus new alternatives. Struct Equ Modeling. 1999;6(1):1–55.

[123] Kline RB. Convergence of structural equation modeling and multilevel modeling. 2011.

[124] Upadyaya K, Eccles JS. How do teachers’ beliefs predict children’s interest in math from kindergarten to sixth grade?. Merrill Palmer Q. 2014;60(4):403–430.

[125] Engle RW. Role of working-memory capacity in cognitive control. Curr Anthropol. 2010;51:S17–S26.

[126] Bergin DA. Social influences on interest. Educ Psychol. 2016 51(1), 7–22.

[127] Ahmed W, Minnaert A, van der Werf G, Kuyper H. Perceived social support and early adolescents’ achievement: The mediational roles of motivational beliefs and emotions. J Youth Adolesc. 2010;39(1):36 10.1007/s10964-008-9367-7 20091215 PMC2796962

[128] Blair C, McKinnon RD, Family Life Project Investigators. Moderating effects of executive functions and the teacher–child relationship on the development of mathematics ability in kindergarten. Learn Instr. 2016;41:85–93. 28154471 10.1016/j.learninstruc.2015.10.001 PMC5283384

[129] Prewett SL, Bergin DA, Huang FL. Student and teacher perceptions on student-teacher relationship quality: A middle school perspective. Sch Psychol Int. 2019;40(1):66–87.

[130] Deary IJ, Strand S, Smith P, Fernandes C. Intelligence and educational achievement. Intelligence. 2007;35(1):13–21.

[131] Karbach J, Gottschling J, Spengler M, Hegewald K, Spinath FM. Parental involvement and general cognitive ability as predictors of domain-specific academic achievement in early adolescence. Learn Instr. 2013;23:43–51.

[132] Lemos GC, Abad FJ, AlmeidaL S, Colom R. Sex differences on g and non-g intellectual performance reveal potential sources of STEM discrepancies. Intelligence. 2013;41(1):11–8.

[133] Lynn R, Vanhanen T. Intelligence: A unifying construct for the social sciences London:Ulster Institut for Social Research; 2012.

[134] Primi R, Ferrão ME, Almeida LS. Fluid intelligence as a predictor of learning: A longitudinal multilevel approach applied to math. Learn Individ Differ. 2010;20(5):446–51

[135] Hembree R. The nature, effects, and relief of mathematics anxiety. J Res Math Educ. 1990;33–46.

[136] Ashcraft MH, Krause JA. Working memory, math performance, and math anxiety. Psychon Bull Rev. 2007; 14(2):243–248. 10.3758/bf03194059 17694908

[137] Hill F, Mammarella IC, Devine A, Caviola S, Passolunghi MC, Szűcs D. Maths anxiety in primary and secondary school students: Gender differences, developmental changes and anxiety specificity. Learn Individ Differ. 2016;48:45–53.

[138] Ma X. A meta-analysis of the relationship between anxiety toward mathematics and achievement in mathematics. J Res Math Educ. 1999;30(5):520.

[139] Mammarella IC, Caviola S, Giofrè D, Borella E. Separating math from anxiety: The role of inhibitory mechanisms. Appl Neuropsychol Child. 2018;7(4):342–53. 10.1080/21622965.2017.1341836 28682117

[140] Marsh HW, O’Mara A. Reciprocal effects between academic self-esteem, self-esteem, achievement, and attainment over seven adolescent years: Unidimensional and multidimensional perspectives of self-esteem. Pers Soc Psychol Bull. 2008;34(4):542–52. 10.1177/0146167207312313 18340036

[141] Ashcraft MH, Kirk EP, Hopko DR. On the cognitive consequences of mathematics anxiety In Donlan C. (Ed.) The development of mathematical skills. (pp. 175–196). East Sussex, Great Britain: Psychology Press 1998

[142] Dowker A. Individual differences in arithmetic: Implications for psychology, neuroscience and education Routledge 2019.

[143] Wallace HM, Baumeister RF. The effects of success versus failure feedback on further self-control. Self Identity. 2002;1(1):35–41.

[144] Baumeister RF, Heatherton TF, Tice DM. When ego threats lead to self-regulation failure: Negative consequences of high self-esteem. J Pers Soc Psychol. 1993;64(1):141 10.1037//0022-3514.64.1.141 8421250

[145] McCormick MP, O’Connor EE, Cappella E, McClowry SG. Teacher–child relationships and academic achievement: A multilevel propensity score model approach. J Sch Psychol. 2013;51(5):611–24. 10.1016/j.jsp.2013.05.001 24060063

[146] Wu JY, Hughes JN. Teacher Network of Relationships Inventory: Measurement invariance of academically at-risk students across ages 6 to 15. Sch Psychol Q. 2015;30(1):23 10.1037/spq0000063 24884450 PMC4254382

